# The impact of a breast cancer diagnosis on marital outcomes and factors associated with divorce and separation

**DOI:** 10.61622/rbgo/2024rbgo60

**Published:** 2024-06-27

**Authors:** Gustavo Werutsky, Mahira Lopes, Rafaela Gomes de Jesus, Antonia Angeli Gazola, Rodrigo Azevedo Pellegrini, Taiane Francieli Rebelatto, Laura von Wallwitz Freitas, Ana Paula Heck, Arthur Ferreira da Silva, Matheus Füehr Rodrigues, Gustavo Gössling, Juliana Giacomazzi, Matheus Soares Rocha, Daniela Dornelles Rosa, Carlos Henrique Barrios, Eduardo Henrique Cronemberger, Geraldo Silva Queiroz, José Bines, Sérgio Daniel Simon, Andre Poisl Fay

**Affiliations:** 1 Latin American Cooperative Oncology Group Porto Alegre RS Brazil Latin American Cooperative Oncology Group, Porto Alegre, RS, Brazil.; 2 Pontifícia Universidade Católica do Rio Grande do Sul Porto Alegre RS Brazil Pontifícia Universidade Católica do Rio Grande do Sul, Porto Alegre, RS, Brazil.; 3 Department of Medical Oncology Hospital São Lucas Pontifícia Universidade Católica do Rio Grande do Sul Porto Alegre RS Brazil Department of Medical Oncology, Hospital São Lucas, Pontifícia Universidade Católica do Rio Grande do Sul, Porto Alegre, RS, Brazil.; 4 Grupo Brasileiro de Estudos em Câncer de Mama Porto Alegre RS Brazil Grupo Brasileiro de Estudos em Câncer de Mama, Porto Alegre, RS, Brazil.; 5 Centro Regional Integrado de Oncologia Fortaleza CE Brazil Centro Regional Integrado de Oncologia, Fortaleza, CE, Brazil.; 6 Associação de Combate ao Câncer em Goiás Hospital Araújo Jorge Goiânia GO Brazil Associação de Combate ao Câncer em Goiás, Hospital Araújo Jorge, Goiânia, GO, Brazil.; 7 Instituto Nacional do Câncer Rio de Janeiro RJ Brazil Instituto Nacional do Câncer, Rio de Janeiro, RJ, Brazil.; 8 Grupo Oncoclínicas São Paulo SP Brazil Grupo Oncoclínicas, São Paulo, SP, Brazil.

**Keywords:** Breast neoplasms, Marital status, Divorce, Separation

## Abstract

**Objective:**

To analyze marital outcomes, divorce or separation, and its association with demographic, socioeconomic, and clinicopathological factors among breast cancer (BC) survivors after 2-years of diagnosis.

**Methods:**

We performed a retrospective analysis of marital status at baseline and at years 1 and 2 of follow-up of women aged ≥ 18 years diagnosed with invasive BC participating in the AMAZONA III (GBECAM0115) study. The BC diagnosis occurred between January 2016 and March 2018 at 23 institutions in Brazil.

**Results:**

Of the 2974 women enrolled in AMAZONA III, 599 were married or living under common law at baseline. Divorce or separation occurred in 35 (5.8%) patients at 2 years of follow-up. In the multivariate analysis, public health insurance coverage was associated with a higher risk of marital status change (8.25% vs. 2.79%, RR 3.09, 95% CI 1.39 - 7.03, p = 0.007). Women who underwent mastectomy, adenomastectomy or skin-sparing mastectomy were associated with a higher risk of divorce or separation (8.1% vs. 4.49%, RR 1.97, 95 CI 1.04 – 3.72, p = 0.0366) than those who underwent breast-conserving surgery.

**Conclusion:**

Women covered by the public health system and those who underwent mastectomy, adenomastectomy or skin-sparing mastectomy were associated with a higher risk of divorce or separation. This evidence further supports the idea that long-term marital stability is associated with a complex interplay between socioeconomic conditions and stressors, such as BC diagnosis and treatment. **ClinicalTrials Registration:** NCT02663973.

## Introduction

Marital status has long been recognized as an important prognostic factor for many cancers. Several studies have also shown that unmarried patients have a higher risk of being diagnosed with breast cancer (BC) at later stages and to die than married women.^(1-3)^Unmarried women with BC living in low socioeconomic status (SES) neighborhoods have a 1.6 times higher risk of dying than married women in high SES neighborhoods.^(4)^

BC diagnosis and treatment can negatively affect survivors’ quality of life, psychological functioning, sexual health, body image, and workability, among other aspects, which may impact their familial relationships of cancer survivors.^(5-7)^ In addition, factors such as cancer’s emotional and financial burden may lead to marital stress, that is, divorce or separation, especially among younger cancer survivors.^(8)^However, some couples reported an improved relationship when coping with BC.^(9)^Studies have also shown an association between psychosocial variables such as open and constructive communication, more social support, supportive coping, and marital adjustment in women and their partners.^(10)^

A population-based study demonstrated that BC was not associated with marital breakdown. However, they described low marital satisfaction within three months of BC diagnosis as a predictor of further marital difficulties.^(11)^ A prospective cohort of 134.435 married women diagnosed with early-stage BC did not demonstrate increased marital dissolution.^(12)^A prospective cohort of patients with BC in Brazil showed that changes in marital status occurred infrequently and were not associated with a return to work 2-years after BC diagnosis.^(13)^

This study aimed to analyze marital outcomes, divorce or separation, and their association with demographic, socioeconomic, and clinicopathological features in a large prospective cohort of women with BC from several regions of Brazil.

## Methods

We performed a retrospective analysis of marital status at baseline and at years 1 and 2 of follow-up of women aged ≥ 18 years diagnosed with invasive BC participating in the AMAZONA III (GBECAM0115) study.^(14)^

The AMAZONA III is a prospective cohort study conducted at 23 sites in Brazil (9 from the southern region, 7 from the southeast, 4 from the northeast, 2 from the center-west, and 1 from the north). The study included women aged 18 years or older with histologically proven invasive BC and clinical stages I–IV (any histology). All consecutive women aged ≥ 18 years who were newly diagnosed with BC between January 2016 and March 2018 were invited to participate. Sociodemographic, clinicopathological, and treatment data were collected at baseline, and patients were followed up for 5 years.^(14)^

Marital status was classified as a dichotomous variable: married or living in a common-law marriage and no formal relationship. In Brazil, common law marriage is a legal framework that considers a couple to be married without formally registering their relationship as civil or religious. The marital status of the women included in the AMAZONA III was collected at baseline, year 1, and year 2 of follow-up during the medical consultation on the patient’s visit to the institution, or by medical chart review.

The eligibility criteria for the marital status analysis were as follows: women with BC diagnosed with a clinical stage (CS) I-III; those who underwent surgery and had available data on marital status at baseline and 2-years after BC diagnosis. Patients with metastatic BC were excluded from this study. Patients who were pregnant at diagnosis, had missing information on marital status at year 2, were lost to follow-up, or died were excluded.

Quantitative variables are described as medians and ranges, whereas categorical variables are described as absolute and relative frequencies. For patients who were married or living in common law at baseline, the risk ratio (RR) of divorce or separation was assessed 2years after BC diagnosis. Univariate Poisson regression analyses with robust variance were used to determine which patient characteristics, tumor features, and BC treatments were associated with changes in marital status. The final multivariate model was obtained using the backward selection method, which began by fitting all independent variables in the model. These variables were considered confounders and were not included in the multivariate model: house income, and employment status.

Next, the variable with the highest p-value was removed from the model and a new model was fitted. This process was repeated until all variables in the model had p-values < 0.20. The significance level was set at 5%. All analyses were performed using SAS version 9.4 (SAS Institute, Cary, NC, USA).

The AMAZONA III study was approved by the Ethics Committee of the Pontifícia Universidade Católica do Rio Grande do Sul (4.811.081). All individuals provided written informed consent for data collection (48573015.5.1001.5330).

## Results

Of the 2974 women enrolled in the AMAZONA III study, 969 were diagnosed with BC at clinical stages I-III. Of these women, 599 were married or living under common law at baseline and were included in the marital status analysis. The sociodemographic and clinicopathological characteristics are presented in tables 1 and 2, respectively. Treatments administered to the included women are shown in table 3.

**Table 1:** Sociodemographic characteristics at baseline

Characteristics	n(%)
Age at breast cancer diagnosis in years - Median (range)	51(25 - 86)
≤50	287(47.91)
>50	296(49.42)
Unknown	16(2.67)
Female Reproductive Status	
Premenopausal/Perimenopausal	275(45.91)
Postmenopausal	317(52.92)
Unknown	7(1.17)
Race	
White	374(62.44)
Black	26(4.34)
Brown	175(29.22)
Yellow	4 (0.67)
Unknown	20(3.34)
Education	
Illiterate	8(1.34)
Did not complete first degree	161(26.88)
Completed first degree	85(14.19)
Completed secondary degree	160(26.71)
Completed superior degree or higher	158(26.38)
Unknown	27(4.51)
Health insurance	
Public	332(55.43)
Private	257(42.90)
Unknown	10(1.67)
Household income per month	
No income - Less than 1 minimum wage (less than R$ 880)	34(5.68)
1 to 2 minimum wages (R$ 880 to R$1760)	136(22.70)
2 to 5 minimum wages (R$ 1760 to R$ 4400)	182(30.38)
More than 5 minimum wages (more than R$ 4400)	89(14.86)
Unknown	158(26.38)
Employment at the time of diagnosis	
Yes	259(43.24)
No	324(54.09)
Unknown	16(2.67)
Number of children	
0	51(8.51)
1	126(21.04)
2	211(35.23)
3 or more	195(32.55)
Unknown	16(2.67)

n - number of patients

**Table 2:** Clinicopathological characteristics at baseline

Characteristics	n(%)
Primary Tumor Histology (biopsy)	
Ductal	493(82.30)
Lobular	36(6.01)
Other	55(9.18)
Unknown	15(2.50)
Tumor grade (biopsy)	
1	111(18.53)
2	280(46.74)
3	127(21.20)
Not tested	39(6.51)
Unknown	42(7.01)
Clinical stage at diagnosis	
I	184(30.72)
II	285(47.58)
III	130(21.70)
Molecular subtype	
HER-2 positive/Luminal B - HER-2 positive	144(24.04)
Luminal A/Luminal B - HER-2 negative	330(55.09)
Triple-negative	78(13.02)
Unknown	47(7.85)

n - number of patients

**Table 3:** Treatments administered for included women

Treatment	n(%)
Adjuvant radiotherapy	
Yes	420(70.12)
No	168(28.05)
Unknown	11(1.84)
Surgery type	
Breast-conserving surgery	362(60.43)
Mastectomy, Adenomastectomy or Skin-sparing mastectomy	215(35.89)
Unknown	22(3.67)
Neoadjuvant chemotherapy	
Yes	401(66.94)
No	196(32.72)
Unknown	2(0.33)
Adjuvant chemotherapy	
Yes	323(53.92)
No	270(45.08)
Unknown	6(1.00)
Adjuvant-targeted therapy	
Yes	10(1.67)
No	589(98.33)
Adjuvant endocrine therapy	
Yes	371(61.94)
No	218(36.39)
Unknown	10(1.67)

n - number of patients

Of the 599 women married or living in common law at baseline, divorce or separation occurred in 31 (5.1%) at 1 year of follow-up and in an additional 4 occurrences, leading to 35 (5.8%) women at 2 years of follow-up. Divorces or separations occurred between 20-39 years for seven patients (20.6%), 40-49 years for ten patients (29.4%), 50-64 years for 11 patients (32.3%), and more than 65 years for six patients (17.7%). Table 4 describes the association of sociodemographic, clinicopathological, and treatment factors with divorce or separation after two years of BC diagnosis. Public health insurance was associated with a higher risk of marital status change compared to private health insurance (8.1% vs. 3.1%, RR 2.61, 95% CI 1.21 - 5.65, p = 0.007). In the multivariate analysis, public health insurance coverage was associated with a higher risk of marital status change (8.25% vs. 2.79%, RR 3.09, 95% CI 1.39 - 7.03, p = 0.007). Additionally, mastectomy, adenomastectomy or skin-sparing mastectomy was associated with a higher risk of divorce or separation (8.1% vs. 4.49%, RR 1.97, 95 CI 1.04 – 3.72, p = 0.0366) compared with breast-conserving surgery.

**Table 4:** Association of sociodemographic, clinical, and treatment factors with divorce or separation after two years of breast cancer diagnosis

Parameter	Univariate analysis					Multivariate analysis^d^				
	n	Divorce or Separation n(%)	Relative Risk^b^	95% CI^c^	p-value	n	Divorce or Separation n(%)	Relative Risk^b^	95% CI^c^	p-value
Age					0.6557					
<=50^a^	287	18(6.27)								
>50	296	16(5.41)	0.86	0.45 to 1.66						
Race					0.2403					
White	374	18(4.81)	0.66	0.34 to 1.28						
Non-white^a^	205	15(7.32)								
Parity					0.9872					
Yes	532	31(5.83)	0.99	0.31 to 3.13						
No^a^	51	3(5.88)								
Employment at the time of diagnosis					0.2601					
Yes	259	12(4.63)	0.68	0.34 to 1.35						
No^a^	324	22(6.79)								
Education					0.6300					
Illiterate - Completed first degree^a^	254	16(6.30)								
Completed second-degree or higher	318	17(5.35)	0.85	0.44 to 1.65						
Patient’s health insurance					0.0070					0.0070
Private^a^	257	8(3.11)				251	7(2.79)			
Public	332	27(8.13)	2.61	1.21 to 5.65		315	26(8.25)	3.09	1.39 to 7.03	
Clinical stage at diagnosis					0.5695					
I	184	11(5.98)	0.78	0.34 to 1.78						
II	285	14(4.91)	0.64	0.29 to 1.40						
III^a^	130	10(7.69)								
Molecular subtype					0.9512					
Luminal A/Luminal B - HER-2 negative	330	16(4.85)	0.95	0.33 to 2.75						
HER-2 positive/Luminal B - HER-2 positive	144	8(5.56)	1.08	0.34 to 3.48						
Triple negative^a^	78	4(5.13)								
Adjuvant radiotherapy					0.4644					
Yes^a^	420	23(5.48)								
No	168	12(7.14)	1.30	0.66 to 2.56						
Surgery type					0.1033					0.0366
Mastectomy, Adenomastectomy or Skin-sparing mastectomy	215	17(7.91)	1.79	0.92 to 3.47		210	17(8.10)	1.97	1.04 to 3.72	
Breast-conserving surgery^a^	362	16(4.42)				356	16(4.49)			
Neoadjuvant chemotherapy					0.5679					0.1680
Yes^a^	196	10(5.10)				189	10(5.29)			
No	401	25(6.23)	1.22	0.59 to 2.49		377	23(69.70)	1.59	0.79 to 3.16	
Adjuvant chemotherapy					0.7422					
Yes^a^	270	15(5.56)								
No	323	20(6.19)	1.11	0.58 to 2.13						
Adjuvant endocrine therapy					0.2925					
Yes^a^	371	19(5.12)								
No	218	16(7.34)	1.43	0.75 to 2.73						

^a^ Reference level; ^b^ Risk of divorce or separation; ^c^ Confidence Interval; ^d^ From 599 patients, 566 had available data for the final multivariate analysis

## Discussion

The notion that husbands abandon their wives upon receiving a diagnosis of BC is not entirely unprecedented, and can create a precarious situation for women grappling with this illness. The emotional and psychological hurdles that accompany a BC diagnosis can engender marital discord, resulting in diminished marital satisfaction and difficulties in acclimating to life post-diagnosis for both cancer survivors and their partner.^(10)^Furthermore, BC treatment can pose threats to fertility and significantly affect the sexual and psychological well-being of young cancer survivors, all of which may contribute to alterations in marital status.^(1,2,15)^

Our study focused on the most significant sociodemographic and cancer-related variables that may have an impact on the complex interplay between divorce and separation following a BC diagnosis. Metastatic BC cases were excluded from the analysis because their prognosis is generally poor and may result in distinct forms of stress for each partner and more pronounced physical symptoms.

At the 2-year follow-up after BC diagnosis, the divorce or separation rate was relatively low, amounting to 5.8%. Neither age nor BC subtype were associated with marital dissolution in women with BC. However, women with public health insurance and those who underwent mastectomy, adenomastectomy or skin-sparing mastectomy had a higher risk of divorce or separation. This finding further supports the notion that long-term marital stability is influenced by socioeconomic conditions and stressors such as undergoing non-conserving surgeries.

Furthermore, our sample’s low number of events and the intricate relationships between variables that could potentially lead to divorce or separation are likely to have played a role in the results. It is worth noting that in the Finnish prospective registry, living with children and having a higher educational level were associated with higher marital stability.^(12)^

According to the Instituto Brasileiro de Geografia e Estatística (IBGE) (https://www.ibge.gov.br), the annual divorce rate in Brazil was 2.6% in 2018 and 2.5% in 2019. Our study found a higher divorce rate than the general Brazilian population during the first year of follow-up (5.1%), but a considerably lower rate during the second year (0.7%). This contrasts with other studies that have shown that marital breakdown at 12 months after diagnosis was not higher in women with BC than in the control group. In that study, the percentage of women reporting dissatisfaction with their current marital relationship was relatively low, between 7.1% and 14.3%, and was generally similar between survivors and controls. Low marital satisfaction within three months of diagnosis predicted further marital breakdown at 12 and 18 months (p = 0.02 at 12 months and p = .01 at 18 months).

Our finding of a 5.8% rate of divorce or separation after 2- years aligns with other published studies. For example, another prospective cohort in Brazil evaluating return to work after a BC diagnosis identified a rate of 4.5% in divorce or separation after 2 years of follow-up. Similarly, a large prospective study of married Finnish women found that 9.7% of the patients were divorced after BC diagnosis after a median follow-up of 17 years. However, in this study, a diagnosis of BC was not associated with a higher risk of marital dissolution compared with women without BC (HR=0.96, 95% CI 0.79–1.17).^(12)^

Previous studies on the impact of BC on marital stability have yielded mixed results. Some studies have not found a significant association between BC and divorce, whereas others have reported an increased risk of divorce among survivors of BC. However, a population-based study showed that cancer survivors, including those with BC, had a higher risk of divorce or separation than the general population (18% vs. 10%; relative risk [RR]=1.77, 95% confidence interval [CI] 1.43–2.19). In this study, married female cancer survivors had a significantly higher risk of divorce or separation than the controls (21% vs. 11%; RR 1.83, 95% CI 1.49–2.25, p<0.001), and the risk was particularly high for patients aged 20-29 years compared to those aged 30-39 years.^(8)^

Our findings indicate that individuals with BC who possess public health insurance exhibit a notably elevated risk of divorce or separation compared with those with private insurance. Although patients covered by the public health system likely possess lower socioeconomic standing, several studies have confirmed this observation, underscoring the importance of lower income as a significant risk factor for divorce.^(16,17)^

In our analysis, BC treatment, specifically mastectomy, adenomastectomy or skin-sparing mastectomy, was revealed as a contributing factor for divorce or separation in a multivariate model. Historically, mastectomy has been associated with depression and alterations in body image, which can influence and impact divorce or separation.^(18,19)^However, we were unable to establish any connection between other aggressive treatments, such as neoadjuvant or adjuvant chemotherapy, and marital dissolution.^(12)^

Our study provides new insights into the factors that influence marital stability in survivors of BC. For instance, we show that the risk of divorce or separation is not constant over time and may be affected by follow-up duration. Additionally, socioeconomic factors such as income and public health coverage may have an impact on marital outcomes. However, it is unclear whether these variables directly influence marital stability or reflect overall socioeconomic status. It is important to consider the social challenges associated with BC diagnosis and treatment, such as financial toxicity and non-return to work, which may impact patients’ socioeconomic status and compromise their marital adjustment. Further research is required to explore these issues.^(5,20,21)^

Our study has some limitations that must be acknowledged. Although data were collected from multiple sites throughout the entire country, 16 out of the 23 sites were situated in the South and Southeast regions of Brazil, which are characterized by higher socioeconomic status and a greater number of patients covered by private health insurance when compared to the North and Northeast regions. The inclusion of more patients from resource-limited areas would strengthen our findings.

## Conclusion

Women covered by the public health system and who have undergone mastectomy, adenomastectomy or skin-sparing mastectomy have been found to be at a higher risk of divorce or separation. This finding provides additional support for the notion that long-term marital stability is influenced by a multifaceted interplay between socioeconomic factors and stressors including BC diagnosis and subsequent treatment.
